# Increase in the titer of lentiviral vectors expressing potassium channels by current blockade during viral vector production

**DOI:** 10.1186/s12868-015-0159-1

**Published:** 2015-05-05

**Authors:** Masayoshi Okada, Naaz Andharia, Hiroko Matsuda

**Affiliations:** Department of Physiology, Kansai Medical University, 2-5-1 Shin-machi, Hirakata, Osaka 573-1010 Japan

**Keywords:** Lentiviral vector, Titer, K^+^ channel, Method improvement, Kir2. 1, TREK-1, Kv1. 4, HERG

## Abstract

**Background:**

High titers of lentiviral vectors are required for the efficient transduction of a gene of interest. During preparation of lentiviral the vectors, the protein of interest is inevitably expressed in the viral vector-producing cells. This expression may affect the production of the lentiviral vector.

**Methods:**

We prepared lentiviral vectors expressing inwardly rectifying potassium channel (Lv-Kir2.1), its dominant-negative form (Lv-Kir-DN), and other K^+^ channels, using the ubiquitously active β-actin and neuron-specific synapsin I promoters.

**Results:**

The titer of Lv-Kir-DN was higher than that of Lv-Kir2.1, suggesting a negative effect of induced K^+^ currents on viral titer. We then blocked Kir2.1 currents with the selective blocker Ba^2+^ during Lv-Kir2.1 production, and obtained about a 5-fold increase in the titer. Higher extracellular K^+^ concentrations increased the titer of Lv-Kir2.1 about 9-fold. With a synapsin I promoter Ba^2+^ increased the titer because of the moderate expression of Kir2.1 channel. Channel blockade also increased the titers of the lentivirus expressing Kv1.4 and TREK channels, but not HERG. The increase in titer correlated with the K^+^ currents generated by the channels expressed.

**Conclusion:**

In the production of lentivirus expressing K^+^ channels, titers are increased by blocking K^+^ currents in the virus-producing cells. This identifies a crucial issue in the production of viruses expressing membrane channels, and should facilitate basic and gene therapeutic research on channelopathies.

## Background

Viral vectors, especially lentiviral vectors, are useful in transducing genes of interest *in vitro*, *in situ*, and *in vivo*. Lentiviral vectors are promising tools for gene therapy because they can transduce postmitotic cells, allow long-lasting expression, and exhibit low toxicity and low oncogenic activity [1]. High-titer lentiviral vectors are required for efficient transduction and time- and cost-savings. Lentiviral vectors are usually produced in 293T cells with the transfection of self-inactivating expression and helper plasmids [2]. Because most lentiviral vectors contain ubiquitously active promoters, the proteins of interest are inevitably expressed in the viral-vector-producing cells, possibly affecting their vector-producing ability.

Potassium (K^+^) channels play pivotal roles in the regulation of cellular excitability and in ion transport in excitable and nonexcitable cells. Abnormalities of the K^+^ channels result in epilepsy, arrhythmia, and disturbances of the immune system [3,4]. For instance, the loss-of-function mutation of strongly inwardly rectifying K^+^ channel 2.1 (Kir2.1), which is expressed in the heart, blood vessels, kidney, and brain, results in type I Andersen–Tawil syndrome [5], and the Kir2.1 knockout phenotype is lethal in mice, indicating the physiological importance of the channel [6-8]. Therefore, lentiviral vectors expressing K^+^ channels, including Kir2.1, may offer possible treatments for some diseases. Studies have shown that the adenoviral expression of Kir2.1 effectively prevents hyperalgesia by suppressing the neuronal activity of dorsal root ganglion cells [9]. Kir2.1 has also been used to modulate the excitability of neural and cardiac cells in experimental animals [10-12].

However, the overexpression of the renal outer medullary K^+^ (ROMK; Kir1.1) channel resulted in neuronal degeneration [13]. In viral-vector-producing cells, their vector-producing capacity might be reduced by the expression of K^+^ channels. In this study, we report that the production of a lentiviral vector expressing Kir2.1 was increased by blocking the cellular Kir2.1 current during its production. Blocking the channel also increased the viral titer when the neuron-specific synapsin I promoter was used. This method was partly applicable to lentiviral vectors expressing other K^+^ channels, depending on the currents in the resting state. This increase in viral titer correlated with the cellular membrane potential, whose major determinant is the K^+^ current, suggesting that the effectiveness of the blockade can be predicted from the membrane potential of K^+^-channel-expressing cells.

## Results

### Blocking the Kir2.1 current increases virus production

**Figure 1:**
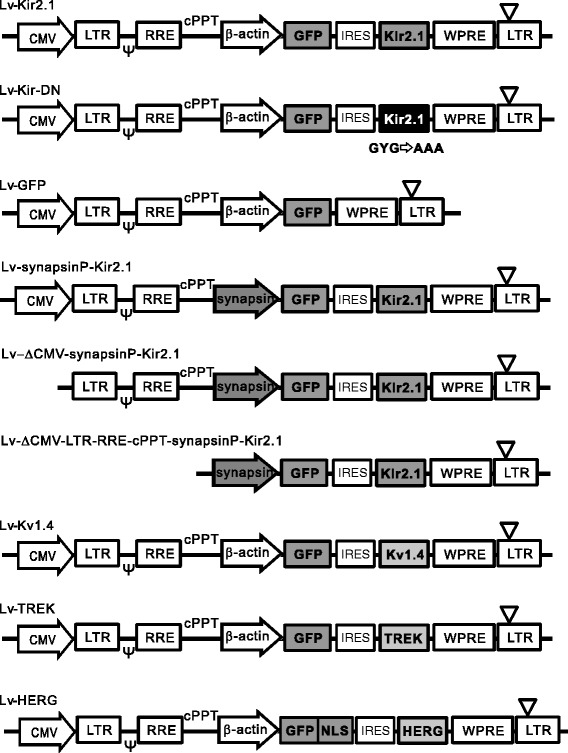
Schematic illustration of lentiviral vectors. Structures of the lentiviral self-inactivating expression plasmids Lv-Kir2.1, Lv-Kir-DN, Lv-GFP, Lv-synapsinP-Kir2.1, Lv-ΔCMV-synapsinP-Kir2.1, Lv-ΔCMV-LTR-RRE-cPPT-synapsinP-Kir2.1, Lv-Kv1.4, Lv-TREK, and Lv-HERG. The upstream CMV promoter was used to transcribe the viral genomic RNA. CMV, cytomegalovirus promoter; LTR, long terminal repeat; *ψ*, RNA packaging signal; RRE, Rev-responsive element; cPPT, central polypurine tract; β-actin, promoter of chick β-actin gene; synapsin, human synapsin I promoter; GFP, green fluorescent protein gene; NLS, nuclear localization signal; WPRE, posttranscriptional regulatory element of woodchuck hepatitis virus; ∇, deletion in the U3 region of the 3′-LTR.

**Figure 2:**
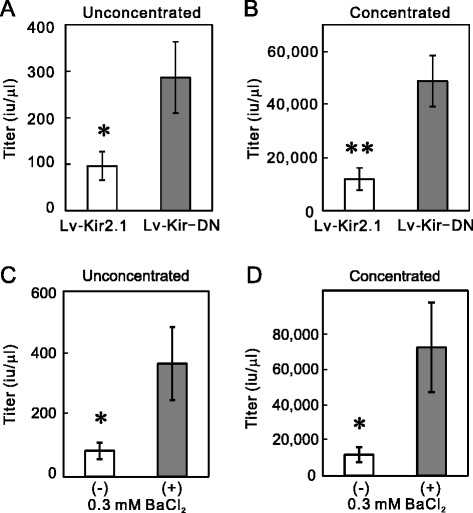
Current-blockade-dependent increase in the titer of Lv-Kir2.1. **(A and B)** The Lv-KirDN titer was higher than the Lv-Kir2.1 titer. The titers were determined with aliquots prepared before (unconcentrated) and after (concentrated) centrifugal concentration. The titers of unconcentrated and concentrated Lv-Kir-DN were both significantly higher than those of unconcentrated and concentrated Lv-Kir2.1 (*n* = 5; *p < 0.05, **p < 0.01, Student’s *t* test). **(C and D)** Blocking the Kir2.1 current increased the titer of Lv-Kir2.1. After plasmid transfection, the 293T cells were incubated in the presence or absence of 0.3 mM BaCl_2_ for 48 h. The addition of BaCl_2_ significantly increased the titers of both the unconcentrated and concentrated Lv-Kir2.1 (*n* = 5; *p* < 0.05, Student’s *t* test).

In our previous study, we prepared lentiviral vectors that bicistronically coexpress Kir2.1 (Lv-Kir2.1) or the dominant-negative form of Kir2.1 (Lv-Kir-DN) and green fluorescent protein (GFP) [14] (Figure 1). In these vectors, we used the chicken β-actin promoter because among all the promoters tested by Okabe et al. [15], it showed the most persistent and highest expression in hippocampal neurons. Because the β-actin promoter is ubiquitously active, the Kir2.1 channels were inevitably expressed in 293T cells during viral production. To test whether their expression affects the viral-vector-producing capacity of the cells, we prepared these two viral vectors and compared their titers before (unconcentrated) and after (concentrated) centrifugal concentration. The titers of Lv-Kir2.1 were significantly lower than those of Lv-Kir-DN in both the unconcentrated (Figure 2A) and concentrated samples (Figure 2B), suggesting that the Kir2.1 current had a negative effect on viral vector production.

**Figure 3:**
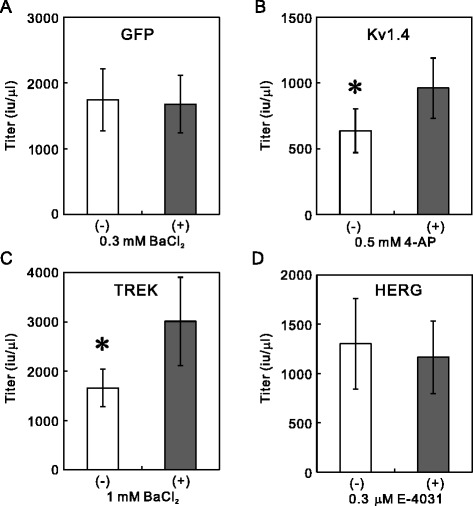
Channel blockers increase titers of Lv-Kv1.4 and Lv-TREK, but not those of Lv-GFP and Lv-HERG. **(A)** Ba^2+^ had no effect on the titer of Lv-GFP. After 293T cells were transfected with Lv-GFP and helper plasmids, they were incubated in the presence or absence of 0.3 mM BaCl_2_ for 48 h. The titers in the unconcentrated aliquots were determined by measuring the number of GFP-positive cell clusters after the transduction to 293T cells (*n* = 5). **(B)** Increase in Lv-Kv1.4 titer. The addition of 4-AP (0.5 mM) significantly increased the titer of Lv-Kv1.4. Because the lentiviral vectors prepared in the presence or absence of blocker were prepared concurrently, the data were analyzed with a paired *t* test (*n* = 5, *p* < 0.05). **(C)** Increase in Lv-TREK titer. Similarly, the transfected 293T cells were incubated in the presence or absence of 1 mM BaCl_2_, and the titers were measured (*p* < 0.05, *n* = 5). **(D)** No increase in Lv-HERG titer. In contrast, the addition of E-4031 did not increase the titer of Lv-HERG (*n* = 8).

To confirm this negative effect, we added a selective Kir2.1 channel blocker, Ba^2+^ (0.3 mM), to the culture medium of the Lv-Kir2.1-producing cells immediately after transfection. We then determined the titers of Lv-Kir2.1 prepared in the presence or absence of Ba^2+^ with the transduction to 293T cells. As expected, the addition of Ba^2+^ significantly increased the lentiviral titers in both the unconcentrated (Figure 2C) and concentrated samples (Figure 2D). Because we prepared the control and Ba^2+^-treated samples concurrently using the same batches of 293T cells and plasmids, the efficacy of plasmid transfection should have been identical in both. To confirm that Ba^2+^ increased the lentiviral titer by blocking the Kir2.1 channels, we prepared a lentiviral vector that only expressed GFP (Lv-GFP; Figure 1) in the presence or absence of 0.3 mM Ba^2+^. The addition of Ba^2+^ had no effect on the titer of the unconcentrated sample (Figure 3A, *n* = 5), suggesting that Ba^2+^ acts on the Kir2.1 channel. Interestingly, the titers of Lv-GFP were higher than those of Lv-Kir2.1 (*p* < 0.05, *n* = 5, Student’s *t* test; see below).

To exclude the possibility that Ba^2+^ affects the infectious ability of the Lv-Kir2.1 particle, we prepared Lv-Kir2.1 without the addition of Ba^2+^ and concentrated it. The infectious ability of the vector was then determined with transduction to 293T cells in the presence or absence of 0.3 mM BaCl_2_. The addition of BaCl_2_ had no effect (Control 12.0 ± 1.7 × 10^4^ iu/μl; BaCl_2_, 10.3 ± 1.8 × 10^4^ iu/μl; *n* = 8, *p* = 0.5, Student’s *t* test). Next, to exclude the possibility that Ba^2+^ affects the stability of the lentiviral particle, Lv-Kir2.1 was incubated in medium with or without 0.3 mM BaCl_2_ for 0, 4, 9, or 24 h at 37°C in a 96-well plate. We then measured the infectious ability of the particle by adding 293T cells to each well and counting the numbers of GFP-positive cell clusters after 48 h. The half-life of the transduction ability of the particles was estimated as the decline in the infectious units. The addition of Ba^2+^ had no effect on the half-lives (control, 9.9 ± 2.5 h; BaCl_2_, 10.2 ± 1.4 h, *n* = 5).

### Correlation between Lv-Kir2.1 titer and Kir2.1 current

**Figure 4:**
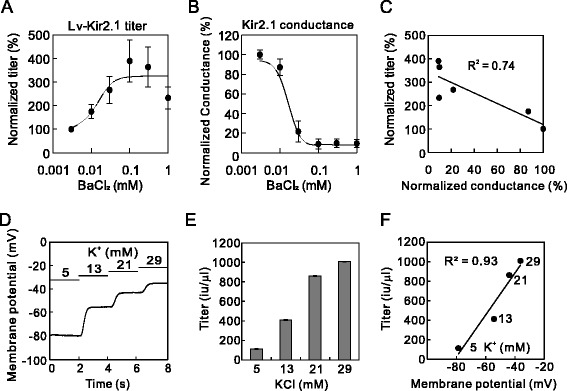
Correlation between Lv-Kir2.1 titer and Kir2.1 current. **(A)** Ba^2+^ concentration–Lv-Kir2.1 titer relationship (*n* = 4). **(B)** Ba^2+^ concentration–Kir2.1 conductance relationship (*n* = 4). These relationships were fitted to the Hill equation (four parameters). **(C)** Correlation between Kir2.1 conductance and lentiviral titers at various concentrations of BaCl_2_. Values were normalized to that of 0.003 mM BaCl_2_ and were fitted by linear regression (*R* ^2^ = 0.74). **(D)** Changes in the membrane potential at higher extracellular K^+^ concentrations. Tyrode’s solution, in which the K^+^ concentration was varied, was perfused sequentially. **(E)** Extracellular-K^+^-concentration-dependent increase in the titer of Lv-Kir2.1. Lentiviral vectors were prepared with medium containing 5, 13, 21, or 29 mM KCl. We determined the lentiviral titers and found significant differences at different extracellular K^+^-concentrations (*p* < 0.0000000001, analysis of variance [ANOVA], *n* = 4). **(F)** Correlation between membrane potential and the Lv-Kir2.1 titer at various extracellular K^+^ concentrations. Values are the means of four experiments and were fitted with linear regression (*R* ^2^ = 0.93).

To confirm the negative correlation between the K^+^ current and lentiviral production, we examined the Ba^2+^ concentration–response relationship for the Lv-Kir2.1 titer and for Kir2.1 conductance, and compared them. To estimate the Ba^2+^ concentration dependence on the titer, we prepared Lv-Kir2.1 in medium containing varying concentrations of Ba^2+^ (Figure 4A) and estimated the half-maximal effective concentration (EC_50_). We also measured the Kir2.1 conductance with varying concentrations of Ba^2+^ with the whole-cell voltage-clamp configuration (Figure 4B) and estimated the half-maximal inhibitory concentration (IC_50_) of Ba^2+^. The EC_50_ of the titer (14 μM) was similar to the IC_50_ of the conductance (16 μM). We then analyzed the correlation between the Lv-Kir2.1 titer and Kir2.1 conductance at various concentrations of Ba^2+^ (Figure 4C) and found a negative correlation (*R*^2^ = 0.74).

To further verify that the K^+^ current decreased the lentiviral titer, we tested the effect of higher extracellular K^+^ concentrations, which reduced the K^+^ gradient. First, we examined the effect of the higher K^+^ concentrations on the whole-cell membrane potential of a Kir2.1-expressing cell with the whole-cell current-clamp method. The resting membrane potential was depolarized according to the extracellular K^+^ concentration (Figure 4D). Lv-Kir2.1 was then prepared in medium containing higher concentrations of K^+^. As expected, the titers of Lv-Kir2.1 increased as the extracellular K^+^ concentration increased (8.9-fold at 29 mM KCl; Figure 4E). The changes in the lentiviral titer correlated well with the membrane potential (*R*^2^ = 0.93; Figure 4F), supporting the negative effect on the production of Lv-Kir2.1.

### Higher transduction to neurons in hippocampal slices

**Figure 5:**
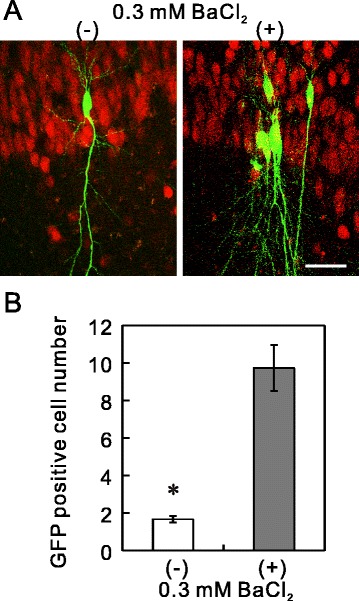
Higher transduction to the hippocampal neurons. **(A)** Concentrated Lv-Kir2.1 vector, prepared in the absence or presence of Ba^2+^, was injected under the same conditions (≈0.05 nl/site) into the extracellular space of the CA1 pyramidal layer in organotypic cultures of rat hippocampus. Seven days after injection, the cultured slices were fixed with 4% paraformaldehyde and immunostained with anti-NeuN antibody and a secondary antibody conjugated with Alexa Fluor 568. The single-plane images were taken with a confocal microscope (218 × 288 μm; FV300, Olympus). Bar, 50 μm. **(B)** Differences in the numbers of GFP-positive cells. Numbers of GFP-positive cells seen within the visual field described above were counted (*n* = 18 and 41, from three independent experiments; *p* < 0.00001, Student’s *t* test).

To test whether target neurons were more effectively transfected with Ba^2+^-treated higher-titer Lv-Kir2.1 than by the control vector, we examined the transduction ability to the hippocampal neurons. We injected concentrated Lv-Kir2.1 vector, prepared in the presence or absence of Ba^2+^, into the CA1 pyramidal layer of a hippocampal organotypic culture. The hippocampal slices were incubated for 7 days, and then fixed and counterstained with an antibody directed against NeuN, a marker protein of neurons (Figure 5A). The number of GFP-positive cells was significantly higher in the Ba^2+^-treated preparation than in the control (Figure 5B).

### Applicability to the vector of neuron-specific synapsin I promoter

**Figure 6:**
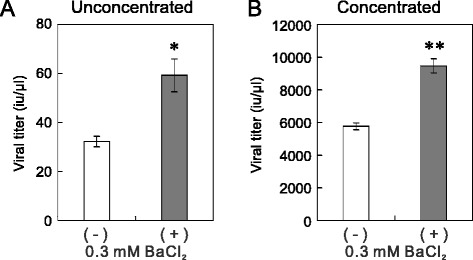
Blocking Kir2.1 current increased the titer of Lv-synapsinP-Kir2.1. **(A and B)** The titer of Lv-synapsinP-Kir2.1 was increased by blocking the Kir2.1 current. The viral vector was prepared in the presence or absence of 0.3 mM BaCl_2_. The titers of unconcentrated **(A)** and concentrated **(B)** viral vectors were measured in host PC12 cells. The addition of Ba^2+^ significantly increased the titers (*p < 0.05, **p < 0.005, Student’s *t* test, *n* = 4).

We then tested whether this method is applicable to a vector that expresses Kir2.1 under the control of the synapsin I promoter, which is considered to act selectively in neuronal cells [16]. We constructed the plasmid Lv-synapsinP-Kir2.1, in which the β-actin promoter was replaced with the human synapsin I promoter (Figure 1). We prepared the lentiviral vector in the presence or absence of 0.3 mM BaCl_2_. The titer of the synapsin-promoter-containing vector was determined with the transduction to PC12 cells because the synapsin I promoter activity is higher in PC12 cells than in 293T cells. Interestingly, the addition of BaCl_2_ increased the titer of Lv-synapsinP-Kir2.1 in both the unconcentrated (Figure 6A) and concentrated samples (Figure 6B). However, the degree of increase (1.8-fold) was smaller than that observed for Lv-Kir2.1 (*p* < 0.05, Student’s *t* test; *n* = 4 for Lv-synapsinP-Kir2.1 and *n* = 5 for Lv-Kir2.1). The titers of Lv-synapsinP-Kir2.1 were lower than those of Lv-Kir2.1, whose titers were determined in 293T cells, because titration using PC12 cells underestimated the titers. For instance, titration with PC12 cells underestimated the titers up to 7.0-fold, even for Lv-Kir2.1 (data not shown).

**Figure 7:**
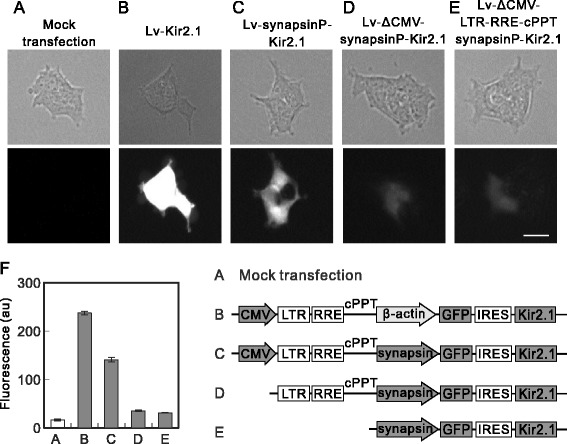
GFP expression from plasmids containing synapsin promoter. **(A, B, C, D, and E)** Expression of GFP with synapsin promoter plasmids. 293T cells were transfected with no plasmid **(A)**, Lv-Kir2.1 **(B)**, Lv-synapsinP-Kir2.1 **(C)**, Lv-ΔCMV-synapsinP-Kir2.1 **(D)**, or Lv-ΔCMV-LTR-RRE-cPPT-synapsinP-Kir2.1 **(E)**. Images were taken after 24 h under a microscope (IX70 Olympus). Bar, 50 μm. **(F)** Summary of GFP expression analyzed with the ImageJ software. The whole-cell area of each cell was manually selected, and the average gray value (*n* = 50, from three independent experiments) is presented as the fluorescence in arbitrary units (au) of the software (*p* < 0.000000001, ANOVA). Note the moderate GFP expression in the Lv-synapsinP-Kir2.1-transfected cells (*p* < 0.005 vs mock transfection; post hoc Student’s *t* test) and its slight expression in the Lv-ΔCMV-synapsinP-Kir2.1- (*p* < 0.01) and Lv-ΔCMV-LTR-RRE-cPPT-synapsinP-Kir2.1-transfected cells (*p* < 0.05). The GFP expression in the two ΔCMV-plasmids-transfected cells was lower than that in the Lv-synapsinP-Kir2.1-transfected cells (*p* < 0.005). Schematic illustrations show the difference in plasmid construction.

The increase in the titer of Lv-synapsinP-Kir2.1 caused by Ba^2+^ suggests that the Kir2.1 channels were moderately expressed in 293T cells. If this is the case, GFP should have also been expressed in the 293T cells because the Lv-synapsinP-Kir2.1 plasmid has a bicistronic expression element, the internal ribosomal entry site (IRES). We examined the GFP expression in 293T cells transfected with plasmid Lv-Kir2.1 or Lv-synapsinP-Kir2.1. As expected, the Lv-synapsinP-Kir2.1-transfected cells expressed GFP 24 h after transfection (Figure 7C), although the expression of GFP was lower than that in the Lv-Kir2.1-transfected cells (Figure 7B).

This finding raises the question: which promoter mediated the expression? There are two possibilities: synapsin promoter might be activated or the transcript derived from the upstream CMV promoter might be translated into Kir2.1 and GFP. To test these two possibilities, we constructed the plasmid Lv-ΔCMV-synapsinP-Kir2.1, in which the upstream CMV promoter was deleted from Lv-synapsinP-Kir2.1, and used it to transfect 293T cells. We observed slight expression of GFP in the cells 24 h after the transfection of the plasmid (Figure 7D), which was clearly higher than that in the mock-transfected cells (compare with Figure 7A). This result suggests that the synapsin promoter was moderately activated in the 293T cells. Furthermore, the expression of GFP from Lv-ΔCMV-synapsinP-Kir2.1 was lower than that from Lv-synapsinP-Kir2.1 (compare Figure 7C and D), suggesting that the transcript from the CMV promoter was also translated to some extent.

To eliminate the possibility of a promoter activity in the fragment containing the long terminal repeat (LTR), central polypurine tract (cPPT), and between them, we deleted that fragment (forming Lv-ΔCMV-LTR-RRE-cPPT-synapsinP-Kir2.1). The GFP expression from Lv-ΔCMV-LTR-RRE-cPPT-synapsinP-Kir2.1 was similar to that from Lv-ΔCMV-synapsinP-Kir2.1 (Figure 7E), indicating the lack of promoter activity in the LTR-RRE-cPPT fragment. The results are summarized in Figure 7F.

### Partial applicability to other K^+^ channels

**Figure 8:**
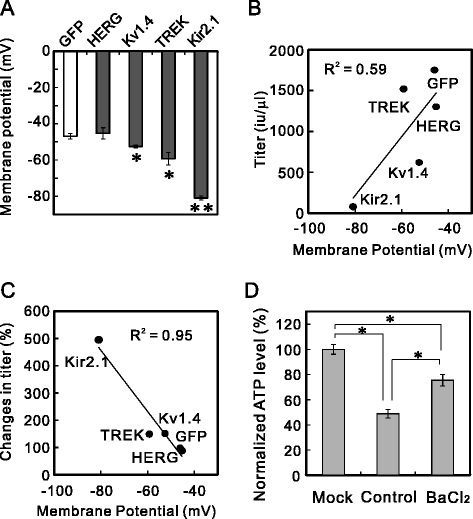
Correlation between the resting membrane potential and lentiviral titer. **(A)** Resting membrane potentials of K^+^-channel-expressing cells. 293T cells were transfected with plasmid Lv-GFP, Lv-HERG, Lv-Kv1.4, Lv-TREK, or Lv-Kir2.1. The membrane potentials of these K^+^-channel-expressing cells were measured in the whole-cell current-clamp mode 24 h after transfection (*p < 0.05, **p < 0.000001 vs Lv-GFP; ANOVA followed by Student’s *t* test, *n* = 5). **(B)** Correlations between membrane potential and the titers of the lentiviral vectors. The titers of the unconcentrated lentiviral vectors encoding GFP, HERG, Kv1.4, TREK, and Kir2.1, prepared without blockers, tended to correlate with the membrane potentials of the 293T cells expressing these channels. Linear regression was used to correlate the data (*R* ^2^ = 0.59). **(C)** Correlation between membrane potential and the increase in the lentiviral vector titer. The membrane potentials of 293T cells expressing GFP, HERG, Kv1.4, TREK, or Kir2.1 correlated well with the percentage change in the lentiviral titer after the addition of the blockers (*R* ^2^ = 0.95). **(D)** Reduction in ATP level and its restoration by Ba^2+^. The ATP levels in 293T cells were measured 48 h after transfection, without harvesting the lentiviral vectors. The ATP levels were normalized to that of the mock-transfected cells (n = 4, *p* < 0.00001; ANOVA followed by Student’s *t* test).

The K^+^ channel family consists of more than 100 subtypes and is the largest family of ion channels. We tested whether the blockade method evaluated here increased the titers of lentiviral vectors expressing other K^+^ channels. We constructed lentiviral vector plasmids that expressed the shaker-type K^+^ channel (Kv1.4), TWIK-related K^+^ channel (TREK-1c), and human ether-a-go-go related gene (HERG) channel (Figure 1). We prepared these lentiviral vectors in the presence or absence of a blocker of each channel and measured the lentiviral titers in unconcentrated aliquots. These viral titers, in samples prepared without a blocker, varied as follows: Lv-Kir2.1 < < Lv-Kv1.4 < Lv-HERG < Lv-TREK < Lv-GFP (Figures 3 and 8B). Interestingly, the channel blockade increased the titers of Lv-TREK and Lv-Kv1.4, but not that of Lv-HERG (Figure 3B, C and D). The degree of increase caused by the addition of the blockers also varied among the channels (Figures 3 and 8C, see below), in the order: Lv-Kir2.1 > > Lv-TREK ≥ LvKv1.4 > Lv-GFP ≥ Lv-HERG, which was almost opposite the order described above.

The lentiviral vector expressing Kv1.4, through which a transient outward K^+^ current (I_A_) flows, was prepared in the absence or presence of a low concentration (0.5 mM) of the I_A_ channel blocker, 4-aminoprydine (4-AP), because higher concentrations of 4-AP affected cell growth (not shown). As shown in Figure 3B, the addition of 4-AP significantly increased the titer of the lentiviral vector to 158.3 ± 12.6% of the control value.

Because the TREK-1c channel is partially blocked by Ba^2+^ [17,18], we added 1 mM BaCl_2_ to the medium of Lv-TREK-producing cells. This addition increased the titer of Lv-TREK to 162 ± 15.8% of the control value (Figure 3C). We also used bupivacaine, which is a more potent blocker of TREK-1 [19], but unexpectedly, it decreased the lentiviral titer. This decrease was attributed to the toxicity of bupivacaine [20] and confirmed by the morphological changes in the cells (not shown). We measured the extent of the blockade with whole-cell patch-clamp recordings and found that 1 mM BaCl_2_ blocked 34.0 ± 7.4% of the bupivacaine (1 mM)-sensitive current expressed in 293T cells (*n* = 3). We also tested the effect of a HERG-specific blocker, E-4031 (0.3 μM), on the Lv-HERG titer, but its addition decreased the titer insignificantly (89.6 ± 11.3%; Figure 3D).

### Correlation between lentiviral titer and membrane potential of cells expressing other K^+^ channels

To understand the variations in the lentiviral titers and their increase by channel blockers, we measured the membrane potentials of 293T cells expressing Kir2.1, GFP, Kv1.4, TREK-1, or HERG. The voltage dependencies of these K^+^ channels differ. For instance, the Kir2.1 channel conducts current at resting and hyperpolarized potentials and is blocked during stronger depolarization. In contrast, the HERG channel only opens during depolarization. Therefore, the amplitude of the K^+^ current in the resting state should differ among these viral-vector-producing cells, and might cause variations in the lentiviral vectors’ titers and the degree of their increase by channel blockers. We measured the membrane potentials, which are largely determined by the K^+^ currents, of 293T cells transfected with these Lv plasmids 24 h after transfection. The membrane potentials were significantly hyperpolarized in the Kv1.4-, TREK-1-, and Kir2.1-expressing cells, in that order (Figure 8A). However, the membrane potential of the HERG-expressing cells was similar to that of the GFP-expressing control cells.

We then assessed the correlation between the membrane potentials of the expressing cells and the viral vector titers. The resting membrane potential tended to correlate with the titers of the lentiviral vectors prepared without blockers (Figure 8B). We next assessed the relationship between these membrane potentials and the degree of increase in the lentiviral titer induced by the channel blockers (Figure 8C). The degree of increase correlated negatively with the membrane potential, i.e., the more hyperpolarized the membrane, the larger the increase in the lentiviral titer, except that the rank order of Kv1.4 and TREK-1 was reversed. Therefore, the difference in the amplitude of the K^+^ current seemed to affect the viral titers and the effectiveness of the blockade.

### Reduced ATP in Lv-Kir2.1-producing cells

The cellular adenosine triphosphate (ATP) level is reportedly critical for virus production [21,22]. The overexpression of the Kir2.1 channel might accelerate ATP consumption by Na^+^/K^+^-ATPase during the intake of K^+^, which is effluxed through the K^+^ channel, reducing the cellular ATP. To clarify the mechanism underlying the changes in the production of Lv-Kir2.1, we measured the cellular ATP levels 48 h after the cells were transfected with the plasmids. The ATP level in the Lv-Kir2.1-producing cells was significantly lower (48.9%) than that in the mock (no plasmid)-transfected cells (Figure 8D). The addition of 0.3 mM Ba^2+^ restored the ATP concentration to 75.5% of that in the mock-transfected cells. Ba^2+^ did not affect the total cell number, which was estimated with microscopic counting and the measurement of lactate dehydrogenase (LDH) activity in living cells lysed with detergent (data not shown).

## Discussion

In this study, several lines of evidence showed that the titers of lentiviruses expressing K^+^ channels were increased when the K^+^ current was blocked during preparation. This evidence is as follows. (1) The titer of Lv-Kir-DN was higher than the titer of Lv-Kir2.1. (2) The addition of Ba^2+^ increased the titer of Lv-Kir2.1. (3) A higher extracellular K^+^ concentration increased the titer of Lv-Kir2.1, which correlated with the membrane potential of the Kir2.1-expressing cells. (4) Channel blockers increased the titers of Lv-Kv1.4 and Lv-TREK, but not that of Lv-HERG. The degree of the lentiviral increase caused by the channel blockers correlated well with the cellular membrane potential. (5) The titers of the lentiviral vectors expressing these K^+^ channels, prepared without blockers, tended to correlate with the membrane potential of the cells expressing each channel. Because the K^+^ current is the major determinant of membrane potential, these results consistently suggest that the viral-vector-producing ability is reduced by an excessive K^+^ current. Therefore, it seems that larger K^+^ currents in the resting state caused lower lentiviral titers without blockers. In addition, the larger currents also resulted in higher degree of the increase in titers with blockers. Membrane potential may be useful in predicting the effectiveness of channel blockers on the lentiviral titer. We anticipate that this method will also be effective for ROMK and epithelial Na^+^ channels, which conduct currents in the resting state. The titers and membrane potentials of Kv1.4 and TREK differed in their ranking, which is probably attributable to the incomplete blockade of the TREK channel by BaCl_2_.

ATP is reportedly a critical factor in the production of vaccinia virus [21] and adenovirus [23]. Burgenner et al. [24] reported a sigmoidal relationship between cellular ATP levels and reovirus production and suggested that there is an “ATP threshold” for virus production. Our data also suggest that reduced ATP plays a causal role in the phenomenon described here. This is the most likely explanation because Na^+^/K^+^-ATPase consumes 55% of the energy in renal cells to maintain the appropriate ion balance [25].

Even when the synapsin I promoter was used, the addition of Ba^2+^ increased the viral vector titer, whereas the degree was lower than that of β-actin promoter. This increase is attributed to both the moderate activation of the promoter and the translation of the transcript from the upstream CMV promoter in 293T cells. The activation of the synapsin I promoter was evident from the slight expression of GFP in the cells transfected with plasmid Lv-ΔCMV-synapsinP-Kir2.1 or Lv-ΔCMV-LTR-RRE-cPPT-synapsinP-Kir2.1, in which the upstream CMV promoter and flanking elements were deleted. The deletion of the CMV promoter also resulted in reduced GFP expression, suggesting that the transcript from the upstream CMV promoter was translated. For these reasons, the titer of Lv-synapsinP-Kir2.1 was increased by the addition of Ba^2+^. This would be the case even if a tissue-specific or inducible promoter were used for the expression of the gene of interest.

Extensive efforts have been made to efficiently produce lentiviral vectors, including the development of packaging cell lines [1,24,26] and the addition of sodium butyrate [27], fructose [28], a kinase inhibitor [29], and caffeine [30]. This study provides another option for increasing the lentiviral titer with a simple method. Such improvements in lentiviral preparation will facilitate basic neuroscience research and the development of clinical gene therapies for channelopathies in the future. Indeed, hippocampal neurons are transduced more effectively with high-titer viral vectors prepared with K^+^ channel blockers.

## Conclusions

Blocking the Kir2.1 current increased the titer of Lv-Kir2.1 without affecting the infectious ability or stability of the viral particles. Hippocampal neurons are more effectively transfected with high-titer viruses than low-titer viruses. Channel blockers also increased the titers of lentiviral vectors expressing some other K^+^ channels, and the extent of these increases correlated with the cell membrane potential. Therefore, it may be possible to predict the effectiveness of channel blockers on lentiviral titers by measuring the membrane potential of the transfected cells. We anticipate that this method will be applicable to other ion channels and will facilitate basic and clinical neuroscience research.

## Methods

### Construction of lentiviral vector plasmids

The construction of the lentiviral self-inactivating expression plasmid is shown in Figure 1. The expression plasmid (CS-CDF-CG-PRE) and the helper plasmids (*gag* and *pol*; *envelope* and *rev*) were provided by Dr. Miyoshi (Riken Tsukuba Institute, Ibaraki, Japan). The downstream CMV promoter of CS-CDF-CG-PRE was replaced with the promoter of the chick β-actin gene (donated by Dr. Takeichi, Riken Kobe Institute) or human synapsin I (donated by Dr. Lois, Caltech). The complementary DNA (cDNA) of GFP was purchased from Stratagene (La Jolla, CA, USA). cDNAs of mouse Kir2.1, rat Kv1.4, human TREK-1c, and HERG were donated by Dr. Jan (UCSF), Dr. Eisenmann (University of Pittsburgh), Dr. Wischmeyer (University of Würzburg), and Dr. Robertson (University of Wisconsin), respectively. We inserted IRES2 between the cDNAs of GFP and the K^+^ channels for their bicistronic expression. Three-point mutations in amino acids G144A, Y145A, and G146A were introduced with PCR to generate the dominant-negative form of Kir2.1. To construct the ΔCMV plasmids, a fragment containing the CMV promoter, LTR, RRE, and cPPT was deleted with restriction enzyme digestion and self-ligation.

### Preparation of the lentiviral vectors

The human embryonic kidney (HEK) 293T cells were maintained in Dulbecco’s modified Eagle’s medium containing 10% fetal bovine serum and 1% penicillin/streptomycin. We transfected the lentiviral self-inactivating expression plasmid and helper plasmid using the calcium-phosphate precipitation method [31]. The viral vectors were pseudotyped with vesicular stomatitis virus G (VSV-G) protein. Five hours after transfection, the 293T cells were washed twice with phosphate-buffered saline (PBS) and incubated in the presence or absence of BaCl_2_ or other blockers. We harvested the media 48 h after transfection and removed the debris by centrifugation (1,000 × *g* for 3 min at 4°C) and filtration (0.8 μm syringe filter). The viral particles were concentrated by centrifugation (58,000 × *g* for 2 h at 4°C) and suspended in 1/400 volume of PBS. Blockers of K^+^ channels, i.e., 0.3 mM BaCl_2_, 0.5 μM 4-AP, 1 mM BaCl_2_, and 0.3 μM E-4031, were added to the media to block the Kir2.1, Kv1.4, TREK-1c, and HERG channel currents, respectively. Lentiviral vectors were simultaneously prepared from the control and current-blocked cells using the same batches of 293T cells and plasmids. The percentage transfection (~70%) was similar in all experiments.

The titers of the lentiviral vectors were determined with the transduction to 293T cells. We prepared sequential 10-fold dilutions series (from 5 μl to 50 pl) for each viral sample in a 96-well plate (triple determinations for each dilution) and added 10,000 293T cells to each well. We counted the numbers of clusters of GFP-positive 293T cells 48 h after transduction. We defined an infectious unit (iu) as the number of transducible viral vector particles per microliter. The titers of the synapsin promoter-containing vectors were determined in pheochromocytoma (PC12) cells (30,000 cells/well), which were incubated for 4 days after transduction. Because PC12 cells seem to be vulnerable to K^+^ channel expression, BaCl_2_ (0.3 mM) was added to the medium during titer determination. The ATP levels were measured with the CellTiter-Glo® kit (Promega, Fitchburg, WI, USA).

### Hippocampal slice culture

We prepared the hippocampal slices (350 μm thickness) from postnatal day 7 Sprague Dawley rats and kept them in culture for 7–13 days in a CO_2_ incubator at 34°C, as described previously in other studies [14,32]. Concentrated Lv-Kir2.1 solution was injected into the extracellular space of the pyramidal cell layer of the slice culture with Femtojet® and Femtotips II ® (Eppendorf, Hamburg, Germany). The slices were incubated for 7 days until fixation. The fixed slices were counterstained with anti-NeuN antibody (MAB377; Millipore, Billerica, MA, USA) and a secondary antibody (Alexa-Fluor-568-conjugated anti-mouse IgG antibody; Molecular Probes, Eugene, OR, USA) to visualize the neurons.

### Electrophysiology

To estimate the IC_50_, we used a 293T cell line, 56-3, that stably expresses Kir2.1 with the lentiviral vector Lv-Kir2.1 [14]. The coverslip on which the 56-3 cells were grown was transferred to a recording chamber on an inverted microscope (Olympus IX70, Tokyo, Japan). The superfusing Tyrode’s solution contained (in mM) 140 NaCl, 5.4 KCl, 0.33 NaH_2_PO_4_, 2 CaCl_2_, 1 MgCl_2_, 5 HEPES, and 5.5 glucose (pH 7.4). To vary the K^+^ concentration of the perfusate, we added 0%, 5%, 10%, and 15% (v/v) 160 mM KCl and 15%, 10%, 5%, and 0% (v/v) 160 mM NaCl to 85% (v/v) Tyrode’s solution, producing various K^+^ concentrations (5, 13, 21, or 29 mM) and equivalent reductions in the Na^+^ concentration from 155 mM. The whole-cell currents were recorded from 293T cells using an Axopatch 200B amplifier (Axon Instruments, Foster City, CA, USA) at 25°C. Patch pipettes pulled from borosilicate glass (Narishige, Tokyo, Japan) were filled with an internal solution containing (in mM) 66 K-aspartate, 71.5 KCl, 1 KH_2_PO_4_, 5 EGTA, 5 HEPES, and 3 K_2_ATP (pH 7.4 adjusted with KOH). The recordings were digitized at 10 kHz and low-pass filtered at 2 kHz. The cells were held at –70 mV, and 400-ms step pulses were applied. The whole-cell conductance was calculated as the slope of the current–voltage relationship between –140 and –100 mV. The membrane potentials of the Kir2.1-, Kv1.4-, TREK-, HERG-, and GFP-expressing cells were measured in the current-clamp mode 24 h after the transfection of each plasmid. The helper plasmids were not transfected to ensure the biosafety of the patch-clamp recorder.

All experiments were approved by the Committee of Gene Recombination Experiments at Kansai Medical University. The animal experiments were performed in accordance with the guidelines of the Physiological Society of Japan and were approved by the Committee on Animal Experiments at our university. All efforts were made to minimize the number of animals used and their suffering. Data are shown as the means ± standard errors of the means (SEM).
